# Club Cell-16 and RelB as Novel Determinants of Arterial Stiffness in Exacerbating COPD Patients

**DOI:** 10.1371/journal.pone.0149974

**Published:** 2016-02-25

**Authors:** Laura E. Labonté, Jean Bourbeau, Stella S. Daskalopoulou, Michele Zhang, Patrick Coulombe, Katie Garland, Carolyn J. Baglole

**Affiliations:** 1 Department of Medicine, McGill University, Montreal, Quebec, Canada; 2 Department of Pathology, McGill University, Montreal, Quebec, Canada; 3 Department of Pharmacology & Therapeutics, McGill University, Montreal, Québec, Canada; 4 Respiratory Epidemiology and Clinical Research Unit, Research Institute of the McGill University Health Center, Montreal, Québec, Canada; 5 Meakins Christie Laboratories, McGill University, Montreal, Québec, Canada; University of Rochester Medical Center, UNITED STATES

## Abstract

**Background:**

Exacerbations of chronic obstructive pulmonary disease (COPD) are acute events of worsened respiratory symptoms that may increase the risk of cardiovascular disease (CVD), a leading cause of mortality amongst COPD patients. The utility of lung-specific inflammatory mediators such as club cell protein-16 (CC-16) and surfactant protein D (SPD) and that of a novel marker of CV outcomes in COPD- RelB- in predicting adverse cardiovascular events during exacerbation is not known.

**Methods:**

Thirty-eight subjects with COPD admitted to the hospital for severe exacerbation were included in this analysis. Clinical, physiological and arterial stiffness measurements were performed within 72 hours of admission; this was followed by measurements taken every 3 days until hospital discharge, then once a week until 30 days after discharge, and then again at 90 and 180 days. Plasma concentrations of inflammatory mediators were measured from peripheral venous blood taken at admission, and at days 15, 30, 90 and 180.

**Results:**

CC-16 and RelB concentrations were increased at day 15 of exacerbations whereas SPD concentrations were decreased. The course of change in CC-16 and RelB levels over time was inversely associated with that of carotid-femoral pulse wave velocity, the gold-standard measure of arterial stiffness. Increases in CC-16 could predict a decreased number of subsequent exacerbations during follow-up.

**Conclusions:**

Lung-specific (CC-16) and novel (RelB) biomarkers are associated with systemic cardiovascular changes over time. CC-16 can predict subsequent exacerbations in subjects with severe COPD and may be an important biomarker of pulmonary and systemic stress in COPD.

## Introduction

Chronic obstructive pulmonary disease (COPD) is a deadly and prevalent lung disease characterized by neutrophilic inflammation, irreversible airflow obstruction and episodes of worsening respiratory symptoms known as exacerbations [1]. Acute exacerbations of COPD are a significant cause of morbidity and mortality [2, 3], contribute substantially to the health care burden for COPD and can accelerate the loss of lung function. Exacerbations are also associated with increased risk of acute cardiovascular (CV) events [4, 5]. Given that cardiovascular disease (CVD) is the second most frequent cause of death in COPD [6, 7], it is imperative to identify biomarkers predictive of increased CV risk in COPD, and in particular, in identifying lung-related biomarkers [8–10] that can predict health outcomes associated with extra-pulmonary consequences of COPD exacerbations.

Exacerbations are associated with acute increases in inflammation in excess of the chronic inflammation that typifies COPD itself, and include significant increases in inflammatory cells as well as mediators. Pulmonary-derived inflammatory mediators that have attracted attention in COPD are club cell protein (CC)-16 and surfactant protein-D (SPD) [10–15]. CC-16 is secreted primarily by non-ciliated bronchiolar club cells [11], with circulating levels largely reflecting protein that is produced in the lungs [16]. CC-16 is thought to play a role in mediating inflammation within the airways [17, 18]. In the ECLIPSE (Evaluation of COPD Longitudinally to Identify Predictive Surrogate Endpoints) study, serum CC-16 levels were stable over time and positively associated with lung function over 3 years [19], a finding further corroborated by Park *et al*. [11] using the Lung Health Study cohort. SPD is produced primarily by type II pneumocytes [20], and is thought to play a role in innate immunity and regulation of surfactant homeostasis in the lung [20, 21]. Circulating SPD levels are inversely associated with lung function in COPD in addition to predicting risk of exacerbation [22, 23]. As the airways become more permeable due to injury, these lung-specific mediators can escape and be detected in the peripheral circulation [24]. Despite their clinical promise, little is known about the course of change in circulating CC-16 and SPD during exacerbations in subjects with severe COPD. Moreover, there is limited evidence about whether these are predictive of other relevant outcomes in COPD, particularly those that are CV-related. In addition to lung-derived biomarkers, another potential indicator of CV outcomes during COPD exacerbations is the nuclear factor-κB (NF-κB) protein, V-rel avian reticuloendotheliosis viral oncogene homolog B (RelB). RelB is an anti-inflammatory component of the NF-κB family that suppresses cigarette smoke-induced inflammation *in vitro* and *in vivo* [25–27]. We have shown that during COPD exacerbations, there is increased peripheral RelB mRNA expression, and this change in expression was inversely associated with and predictive of systolic blood pressure in COPD [28]. While there is some experimental evidence that RelB modulates aspects of CV function [29–31], no information currently exists on RelB in the context of CVD during COPD exacerbations.

We hypothesized that systemic alterations in CC-16, SPD and RelB would be associated with increased carotid femoral-pulse wave velocity (cf-PWV), the gold standard measure of central artery stiffness and powerful predictor of CV risk, and that this risk would increase with escalating exacerbation frequency. Our data support that lung-specific and novel inflammatory mediator expression may be reflective of a relationship between CV risk and COPD.

## Materials and Methods

### Study Subjects

Subjects with a confirmed diagnosis of COPD and a known history of CVD or CV risk factors were recruited upon admission to the Montreal Chest Institute for COPD exacerbation between August 2012–2013. Subjects were assessed within 48 ± 24 hours of hospital admission, and then every 72 ± 24 hours following this until discharged. Subjects were then assessed (see Measurements below) once a week up to 30 days, and then at days 90 and 180. Exclusion criteria included: 1) acute medical conditions other than COPD exacerbations (cancer, ischemic heart event, etc.); or 2) unwillingness/inability to provide informed consent. Subjects were considered to be “ever smokers” if they had smoked cigarettes at some point in their lives. Smoking pack-years were determined by calculating the average number of cigarettes smoked per day per year of smoking.

### Measurements

Subjects were assessed for post-bronchodilator spirometry, venous blood sample collection and arterial stiffness/pressure, within 48 ± 24 hours of hospital admission and then every 72 ± 24 hours until discharge. Subjects were then assessed once a week up to 30 days since initial assessment, and then at days 90 and 180 from initial assessment. Ethics approval was obtained from the McGill University Faculty of Medicine Institutional Review Board (A04-M20-12B). All participants provided written informed consent.

### Arterial stiffness measurement by cf-PWV

Increased central artery stiffness independently predicts risk of CV events and all-cause mortality [32–34]. Carotid-femoral pulse wave velocity (cf-PWV), considered the gold standard measure of arterial stiffness, is safe and non-invasive [35, 36], and independently predicts CV risk and mortality [32, 34, 37]. Arterial stiffness, assessed by cf-PWV, was measured in duplicate at rest using the SphygmoCor system (AtCor Medical, Sydney, Australia). Prior to measurements, subjects rested for at least 10 minutes in a supine position and refrained from speaking. Cf-PWV was determined using arterial waveforms measured using a hand-held tonometer (SPC-301; Millar Instruments, Houston, TX, USA) applied to the surface of the skin overlying the carotid and femoral arteries and gated using a 3-lead electrocardiogram. By measuring the distance between the two recording sites (carotid and femoral arteries), PWV was calculated [PWV = distance (m)/transit time (s)] [38].

### Inflammatory mediator analyses

Peripheral venous blood samples were collected from study participants at the first assessment time point, and then at days 15, 30, 90 and 180. Samples were immediately centrifuged for plasma isolation, which was then aliquoted and stored at -80°C until biomarker analysis. Concentrations of circulating CC-16 and SPD (BioVendor Laboratory Medicine, Modrice, Czech Republic) and RelB (MyBioSource Inc., San Diego, CA) were measured in triplicate using commercially available enzyme-linked immunosorbent assay kits according to the manufacturer’s instructions.

### Statistical Analysis

Analyses were performed using SAS version 9.3 (SAS Institute. Inc., Cary, North Carolina). To decrease inter-subject variation, values are presented as absolute changes (1ng/ml for inflammatory mediators or 1m/s for cf-PWV) from initial assessment; if initial values were not obtained, measurements collected at the next available time point were used to anchor absolute change calculations. For mean comparisons, two-tailed T-tests (normal distribution) or Wilcoxon signed-rank tests (non-normal distribution) were used; Chi-squared tests were used for dichotomous variables. A p-value of 0.05 or less was deemed statistically significant. Mixed-effects linear models were used to estimate the association between increases in the absolute change in circulating inflammatory mediator concentrations and cf-PWV or the number of exacerbations over time. The model included random intercepts to capture individual-specific change in levels and a spatial power correlation structure [spl (pow)] to account for varied time intervals between repeated measurements, adjusted for age, sex, and FEV_1_ (% predicted). The relative risk of exacerbations during follow up based on increases in the absolute change in inflammatory mediator concentrations over time were estimated using Poisson regression models.

## Results

### Clinical characteristics of subjects at exacerbation

Table 1 shows the baseline characteristics and clinical measurements of subjects during hospital admission for acute COPD exacerbation. A total of 38 subjects were assessed with a mean age of 71.7 years (range was 55–88 years); slightly more than half of the subjects were male. Subjects had severe airflow obstruction and an extensive smoking history, with all of them being ever-smokers.

**Table 1:** Characteristics and clinical measurements at initial assessment during exacerbation in subjects with COPD (n = 38).

**Characteristics**	
Age, mean (± SD), years	71.7 (8.23)
Men (%)	55.3
Smoking status (%)	
Former	81.6
Current	18.4
Smoking pack-years, mean (± SD)	55.6 (31.9)
Use of long-term oxygen therapy (%)	23.7
Mean number of exacerbations reported in previous year	2.55
**Clinical measurements**	
FEV_1_% predicted, mean (± SD)	34.0 (14.9)
FEV_1_ mean (± SD), (L)	0.777 (0.320)
FEV_1_/FVC, mean (± SD)	0.484 (0.142)
Body mass index, mean (± SD), kg/m2	25.7 (5.47)
Systolic blood pressure, mean (± SD), mmHg	125.9 (17.1)
Diastolic blood pressure, mean (± SD), mmHg	65.6 (12.4)
Carotid-femoral pulse wave velocity, mean (± SD), m/s	11.6 (2.68)
**CVD/CV risk factor, %**	
Hypertension	47
Angina	16
High cholesterol	13
Coronary artery disease	11
Others	61
**Inflammatory mediator concentrations**	
CC-16, mean (± SD), ng/mL	7.29 (4.33)
SPD, mean (± SD), ng/mL	234.69 (166.24)
RelB, mean (± SD), ng/mL	1.87 (1.52)

SD: standard deviation, FEV1: forced expiratory volume in 1 second, FVC: forced vital capacity, CC-16: club cell-16, SPD: surfactant protein D, RelB: V-rel avian reticuloendotheliosis viral oncogene homolog B.

### Absolute changes in inflammatory mediator concentrations over time

**Fig 1:**
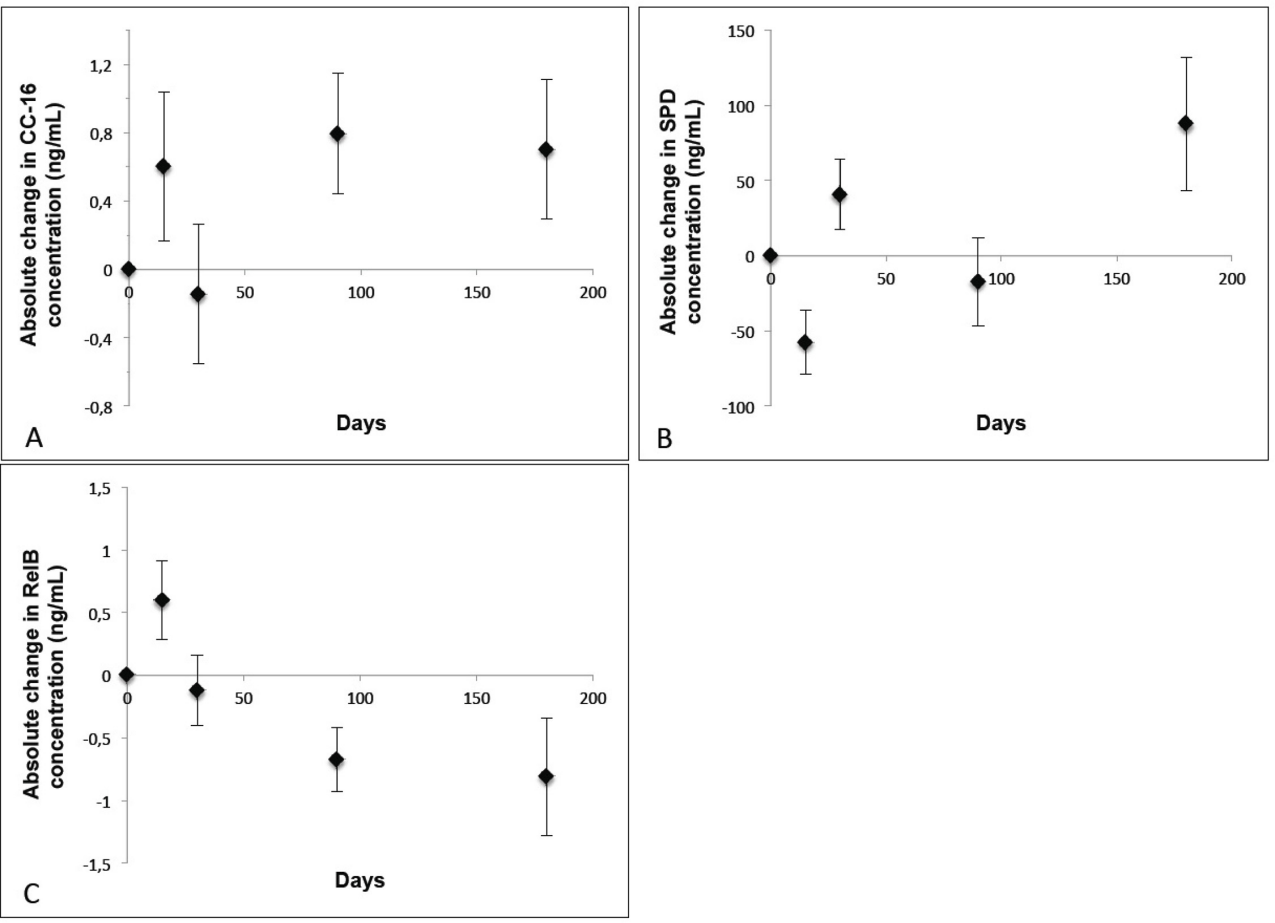
Mean absolute change in inflammatory mediators (ng/mL) (± standard error of the mean) over time. Panel A shows the course of change in CC-16 over time compared to CC- 16 concentrations measured at initial assessment, panel B shows the course of change in SPD over time compared to SPD concentrations measured at initial assessment, and panel C shows the course of change in RelB over time compared to RelB concentrations measured at initial assessment. At day 15, concentrations of CC-16 and RelB were elevated compared to initial concentrations, whereas SPD concentrations were decreased. By day 30, both CC-16 and RelB were lower than initial levels but the level of SPD was increased. The directions of the absolute change in inflammatory mediators varied at the day 90 and 180 time points; all values were different from those at initial assessments.

Table 1 also shows the mean concentrations of CC-16, SPD and RelB at first assessment, which was during the hospitalization phase. The absolute course of change in the concentration of the 3 inflammatory mediators over time compared to those measured during the initial assessment is shown in Fig 1. At day 15, concentrations of CC-16 (Fig 1A) and RelB (Fig 1C; S1 Fig) were increased compared to the initial levels, whereas the levels of SPD (Fig 1B) were decreased. By day 30, both CC-16 and RelB were decreased compared to their initial levels but the level of SPD was increased. The directions of the absolute change in inflammatory mediators varied at the day 90 and 180 time points; all values were different from those at initial assessments as shown in Fig 1. As the course of change in CC-16 was similar to that of RelB during exacerbation, we assessed the relationship between them over time. A 1-unit (1 ng/mL) increase in RelB did not produce a significant change in either CC-16 or SPD (β = -0.027, 95% confidence interval (CI) -0.143 to 0.088, p = 0.64; and β = -5.967, 95% CI -13.617 to 1.683, p = 0.125, respectively).

### Associations between increased inflammatory mediator concentrations and changes in cf-PWV over time

**Fig 2:**
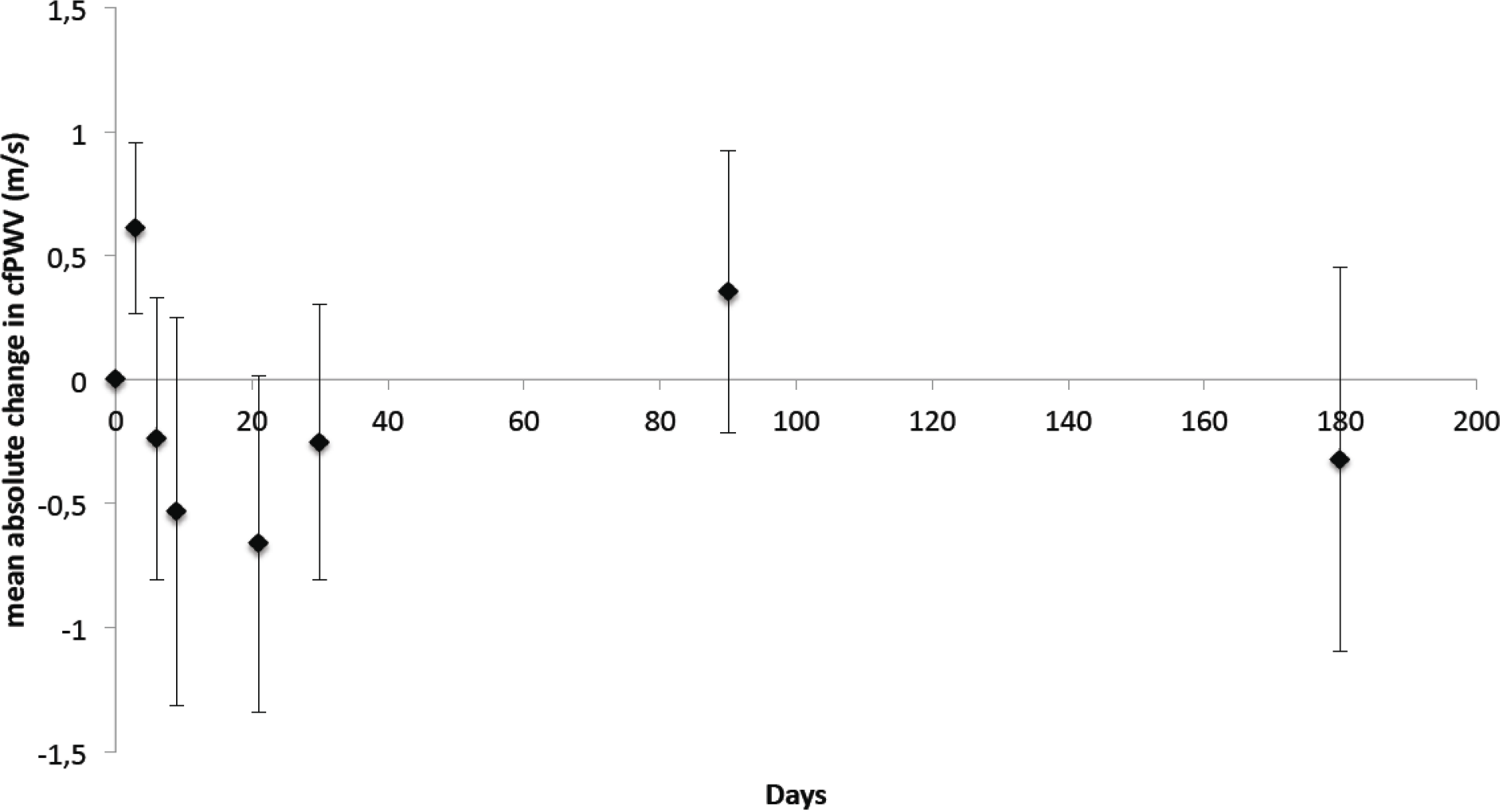
Absolute course of change in cf-PWV (m/s) (± standard error of the mean) over time. Mean absolute change in cf-PWV (m/s) over time relative to initial cf-PWV measurement taken within 48 ± 24 hours of hospital admission as assessed using applanation tonometry. Cf-PWV increased acutely at day 3 after initial assessment, and declined thereafter, remaining lower than at initial assessment until day 30. At day 90, there was a slight increase in cf-PWV compared to day 30, but at day 180, cf-PWV returned to the levels measured at day 30.

**Fig 3:**
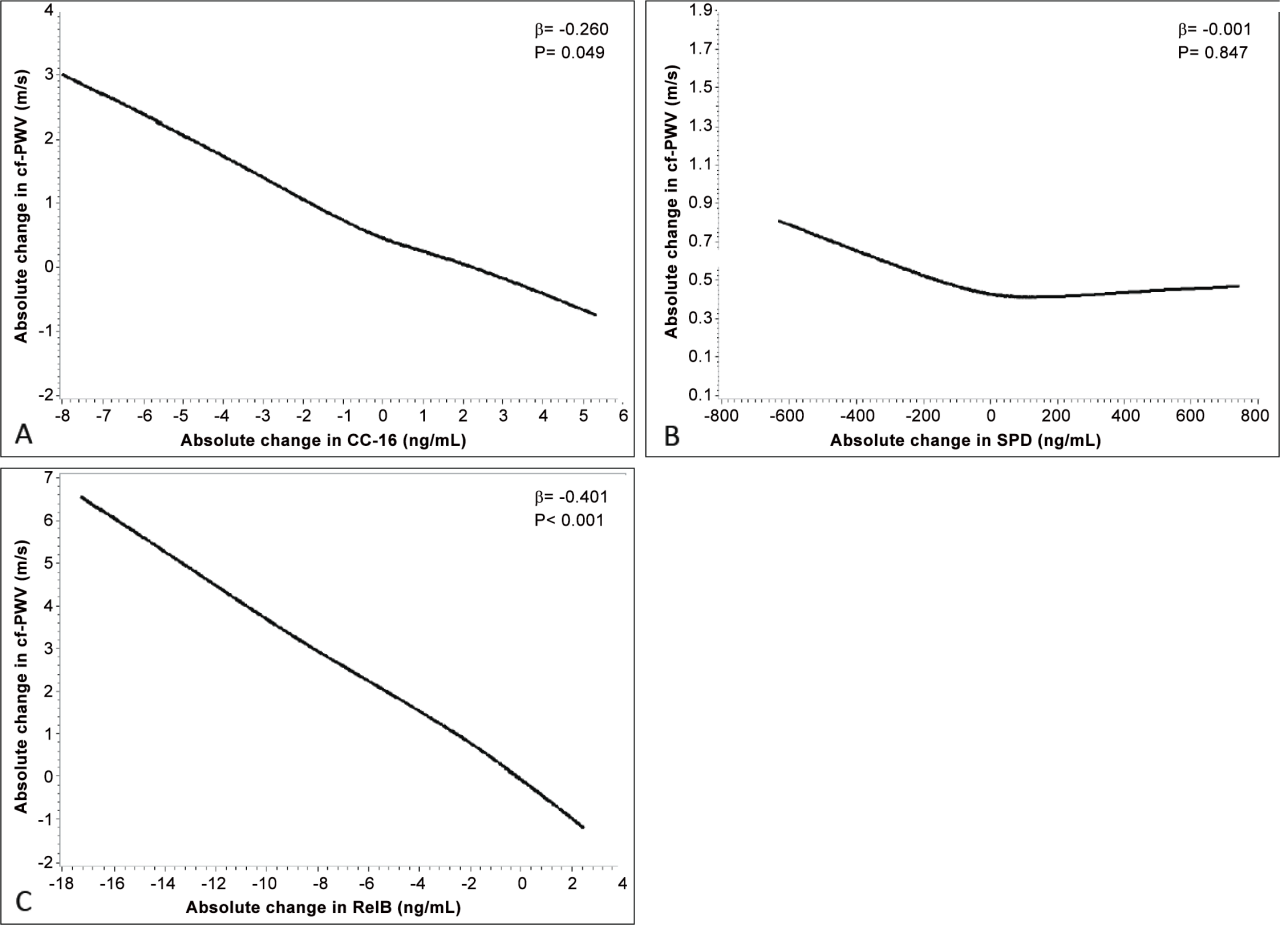
Relationship between the absolute course of change in inflammatory mediator concentrations (ng/mL) over time to that of changes in cf-PWV (m/s). Panel A shows the relationship between the absolute change in CC-16 and cf-PWV over time, panel B shows the relationship between the absolute change in SPD and cf-PWV over time, and panel C shows the relationship between the absolute change in RelB and cf-PWV over time. There was a strong statistically significant negative relationship between the absolute change in CC-16 and cf-PWV as measured over time, as well as between RelB and cf-PWV (p<0.05). There appeared to be a negative relationship between the absolute change in SPD and cf-PWV over time, although this was not found to be statistically significant. Mixed-effects linear models adjusted for age, sex, forced expiratory volume in 1 second % predicted and days since initial exacerbation were used to estimate these relationships. β values show the resulting change in cf- PWV in m/s with a 1-unit (ng/mL) increase in an inflammatory mediator.

Fig 2 shows the mean course of change in cf-PWV over time. After initial assessment, cf-PWV appeared to have acutely increased at day 3 during exacerbation, and then gradually declined up to day 30. Between days 3 and 30, cf-PWV measurements remained lower than that measured at initial assessment. At day 90, there seemed to be a slight elevation in cf-PWV measurements, while cf-PWV appears to have declined at day 180. Fig 3 shows the relationships between the absolute course of change in inflammatory mediators and cf-PWV We found statistically significant inverse associations between increases in the absolute course of change in CC-16 concentrations and the absolute course of change in cf-PWV over time (Fig 3A) as well as between increases in the absolute course of change in RelB concentrations and the absolute course of change in cf-PWV over time (Fig 3C). There was a trend for an inverse association between SPD and cf-PWV over time (Fig 3B). Using multivariable analysis that included all 3 mediators, we found that increases in the absolute concentration of CC-16 and RelB resulted in similar decreases in cf-PWV independent of changes in systolic blood pressure over time (β = -0.320, 95% CI-0.569 to -0.071, p = 0.013; and β = -0.363, 95% CI -0.514 to -0.212, p<0.001, respectively), while that of SPD remained non-significant.

### Increased inflammatory mediator concentrations over time and subsequent exacerbations

Twenty-eight subjects experienced one or more exacerbation during 6 months of follow-up. Table 2 shows the relative risks between increases in the absolute change in inflammatory mediator concentrations over time and subsequent exacerbations. We found that increased CC-16 (by 1 ng/ml) was associated with a 16% reduction in subsequent COPD exacerbations in the next 180 days. There were no significant associations between increases in SPD or RelB concentrations and subsequent exacerbations over time.

**Table 2:** Relative risks of increasing inflammatory mediator concentrations over time and subsequent exacerbations.

1 unit (ng/mL) increase in the absolute change in inflammatory mediators over time	Number of exacerbations during 6 months of F/U	
CC-16	0.84 (0.75–0.95)	0.004*
SPD	0.99 (0.99–1.01)	0.894
RelB	1.02 (0.93–1.11)	0.717

Poisson regression models were used to estimate relative risks of exacerbations. RR adjusted for baseline age, sex, and FEV1% predicted.

*Denotes statistical significance of p<0.05.

CC-16: club cell-16, SPD: surfactant protein-D, RelB: V-rel avian reticuloendotheliosis viral oncogene homolog B, CI: confidence interval, RR: relative risk.

## Discussion

In this study, we show novel evidence that CC-16 may be a potential biomarker that links pulmonary inflammation to arterial stiffness, a composite measure of vascular health in COPD. Additionally, we provide further evidence to support that RelB may mediate vascular outcomes in COPD. However, we did not find any associations between these outcomes and SPD. Finding biomarkers of patient-relevant outcomes is an emerging goal of COPD research, with lung-specific proteins being recognized as one of the most useful strategies in terms of identifying either disease-specific or disease activity-specific markers for COPD (8). Despite the significant impact that CV comorbidity has on COPD, there have been no studies relating lung-specific inflammatory mediators to CV outcomes or CV risk in patients with COPD.

To date, specific lung-derived mediators, *i*.*e*., CC-16 and SPD have been identified as potential biomarkers in COPD [11, 12, 22, 23]. Of these, CC-16 has emerged as a possible mediator of lung function in COPD, where reduced CC-16 levels are associated with accelerated decline in lung function over time as well as COPD progression [11, 12]. CC-16 has also been described in other respiratory conditions, whereby decreased circulating levels are associated with obliterative bronchiolitis [39], asthma [40] and smoking [41]. The ECLIPSE data have shown that repeated measures of CC-16 are stable over time [23], and a recent randomized clinical trial showed that CC-16 levels can be modulated via treatment with salmeterol/fluticasone [42]. As a result, CC-16 represents an attractive biomarker reflective of disease outcomes in COPD. In our study, we report for the first time a relationship between CC-16 and CV function in COPD patients, where increased circulating CC-16 is associated with decreased arterial stiffness. While our study did not allow us to examine the mechanisms responsible for this, we hypothesize that it may involve the ability of CC-16 to inhibit phospholipase A2 (42). Phospholipase A2 can modify low-density lipoproteins, leading to increased uptake by macrophages, a feature in pre-atherosclerotic arterial wall that may lead to low-density lipoprotein modification, foam cell formation and inflammation to promote atherogenesis [43]. It is also possible that CC-16 may act directly on the vascular endothelium or regulate other downstream effectors that lead to increased stiffening of the vessel walls, a notion that has yet to be explored.

We also found that increases in CC-16 could predict lower risk of subsequent exacerbations during follow-up, a finding not observed in the ECLIPSE study [12]. This discrepancy may be due to inherent differences between the populations studied. Our sample of subjects consisted of severely ill patients with advanced disease (GOLD stages 3–4) that have an elevated CV risk and likely reflect the frequent-exacerbator phenotype of COPD patients [44]. Furthermore, the ECLIPSE population included subjects with moderate airflow obstruction who were not all frequent-exacerbators nor were they at elevated CV risk. Patel et al. [4], recently found that increased exacerbation frequency is associated with elevated cf-PWV. Thus, taken together, we hypothesize that decreased CC-16 may reflect increased exacerbation frequency, which in turn, could lead to increased arterial stiffness and increased CV susceptibility. Although there was considerable inter-subject variability in cf-PWV (Fig 2), there was by 3 days post-baseline assessment an acute rise in cf-PWV across all patients. This supports observations by Donaldson and colleagues [5] that COPD exacerbation is associated with a significant increase risk of myocardial infarction within the 5-day period following an exacerbation. Thus, our data present an interesting and highly clinically-relevant mechanism. While further research is needed to understand the role of CC-16 in COPD, our study provides interesting and novel data on CC-16 in COPD exacerbation and CV risk.

The importance of RelB, either in relation to the lung or to obstructive airway diseases such as COPD is only just starting to become recognized [27, 28, 31, 45]. RelB, a member of the NF-κB family, has been identified as an effective suppressor of cigarette smoke-induced inflammation. RelB is constitutively expressed in human lymphocytes and dendritic cells [46], suppresses cytokine production in lung epithelial cells [47] and is important for thymus development and T cell function [48, 49]. There is also growing evidence that RelB may be able to modulate endothelial function. Experimentally, RelB has been associated with balloon catheter injury in the rat carotid artery [29], and its expression can be modulated via treatment with DETA-NONOate-a nitric oxide donor [30]. Our group also showed that RelB is expressed in endothelial cells and such expression can suppress pulmonary ICAM-1 levels in response to smoke [31]. We were the first to show that peripheral RelB expression in COPD subjects is inversely associated with systolic blood pressure at exacerbation [28]. In the current study, we showed that circulating RelB protein concentrations are inversely associated with cf-PWV over time. Taken together, it seems plausible that RelB is an important modulator of the endothelium, and hence, vascular function in COPD patients. We found no association between the course of change in RelB and that of either CC-16 or SPD over time, suggesting that RelB may not directly regulate their expression. This also suggests that different biological pathways may be involved in mediating changes in cf-PWV that involve CC-16 and RelB, or it may suggest that both these mediators have a common upstream regulator. Further studies to better examine the mechanistic role of RelB in CVD and COPD could reveal a novel pathway for intervention.

This study has a number of strengths, but is not without its limitations. One of the major strengths of our study is the focus on a “high-risk” and clinically-relevant COPD patient population. Frequent exacerbators are known to be at elevated CV risk [44], and as such, better understanding this association is important and could improve health outcomes for patients. Our measurements of inflammatory mediators at several points over time provided new information on their course of expression. This allowed us to better assess their relationship to patient-relevant outcomes, including exacerbation frequency and arterial stiffness. Our study also has certain limitations including the relatively small sample size that may have resulted in insufficient power for our analyses due to high inter-individual variation. A second limitation is that we were not able to assess subjects before their exacerbation or prior to receiving treatment with corticosteroids or systemic antibiotics, which may have altered the values of both the mediator and arterial stiffness. CC-16 and SPD expression for example can be modulated by systemic corticosteroids [22, 42], and as such, treatments may have influenced the expression levels obtained in this study. We also did not have information pertaining to other medications- such as anti-hypertensive drugs- that may have impacted our results. For a few subjects, we were not able to get measurements at the initial assessment, and as such had to “gate” absolute change calculations to the second assessment. This may have caused us to miss certain changes that could have occurred in that time. In the future, it would be useful to know the course of change in the mediators (and arterial stiffness) during the first few days and even weeks of exacerbations, as this could provide important information on their course of change and relevance to exacerbation and allow for a more robust assessment. Finally, not having stable-state measurements on inflammatory mediators does not allow us to fully assess the significance of changes measured over time with exacerbation. Stable-state RelB protein measurements have not yet been reported in COPD patients, and so measuring these in future research would be a worthy objective.

Our study serves as an important step towards identifying biomarkers in subjects with frequent exacerbations that relate pulmonary inflammation to CV function, and CC-16 may represent such a marker. Moreover, with the potentially modifiable expression of CC-16 in COPD patients, further research should address whether modulation of CC-16 can lead to changes in arterial stiffness, which could ultimately decrease susceptibility to CV events. In this study we also show that RelB expression is related to arterial stiffness, which points to another potential pathway for the modulation of endothelial and vascular function in COPD. Although the direction of absolute change in CC-16 and RelB diverged at later time points in our study (days 90 and 180), during the course of exacerbation and the time immediately thereafter, both followed the same trajectory of change as cf-PWV. CC-16 and RelB likely have different and perhaps unrelated roles in modulating cf-PWV, and further mechanistic studies are now needed to help elucidate the pathways linking CC-16 and RelB to these outcomes. A better understanding of these outcomes will bring us one step closer to determining their value as biomarkers of patient-relevant CV outcomes in COPD.

## Conclusions

Changes in the expression of club cell-16, a lung specific inflammatory mediator, and RelB, a potential biomarker of cardiovascular function, during and subsequent to COPD exacerbations that require hospital admission can determine changes in arterial stiffness. CC-16 can predict exacerbation frequency, and may represent an important biomarker of pulmonary and cardiovascular function in severe COPD patients.

## Supporting Information

S1 Fig Box plot showing mean absolute change in RelB concentrations (ng/mL) over time as compared to first assessment. Bars represent the maximal and minimal values obtained.
(PPTX)
