# Supplementary figures and images for: Club Cell-16 and RelB as Novel Determinants of Arterial Stiffness in Exacerbating COPD Patients
Source: PLoS One. 2016 Feb 25;11(2):e0149974. doi: 10.1371/journal.pone.0149974 (PMC4767820; doi:10.1371/journal.pone.0149974)

## Slide 1
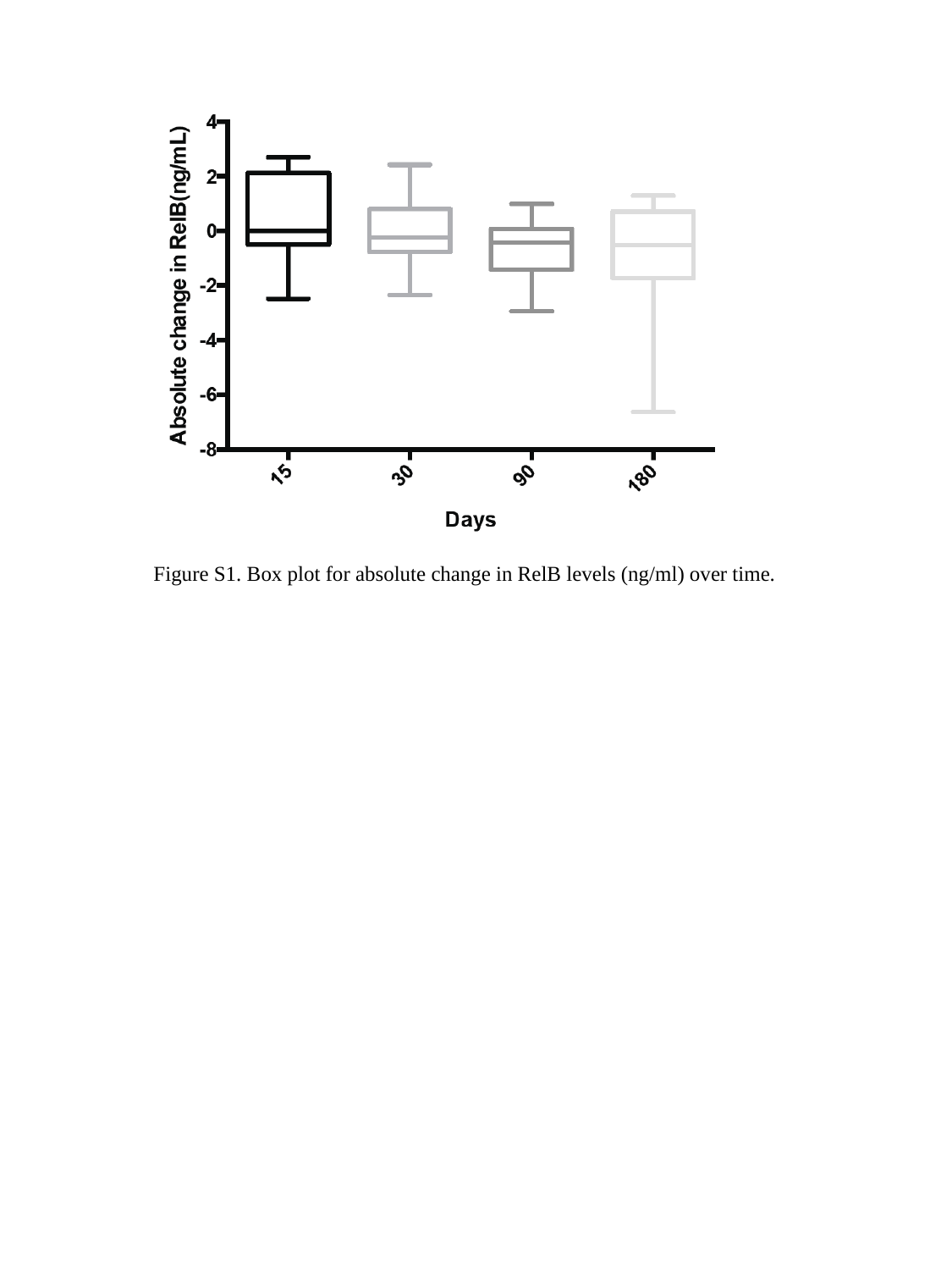

Figure S1. Box plot for absolute change in RelB levels (ng/ml) over time.

Supplement: S1 Fig — Bars represent the maximal and minimal values obtained. (PPTX) [file pone.0149974.s001.pptx]
